# Statin therapy causes gut dysbiosis in mice through a PXR-dependent mechanism

**DOI:** 10.1186/s40168-017-0312-4

**Published:** 2017-08-09

**Authors:** Jose A. Caparrós-Martín, Ricky R. Lareu, Joshua P. Ramsay, Jörg Peplies, F. Jerry Reen, Henrietta A. Headlam, Natalie C. Ward, Kevin D. Croft, Philip Newsholme, Jeffery D. Hughes, Fergal O’Gara

**Affiliations:** 1 Human Microbiome Programme. School of Biomedical Sciences. Faculty of Health Sciences, Curtin University, Perth, WA Australia; 2 Curtin Health Innovation Research Institute (CHIRI), Curtin University, Perth, WA Australia; 3 School of Biomedical Sciences, Faculty of Health Sciences, Curtin University, Perth, WA Australia; 4 School of Pharmacy, Faculty of Health Sciences, Curtin University, Perth, WA Australia; 5 Ribocon GmbH, Fahrenheitstr 1, 28359 Bremen, Germany; 6 BIOMERIT Research Centre, School of Microbiology, University College Cork, Cork, Ireland; 7 School of Medicine and Pharmacology, The University of Western Australia, Perth, WA Australia

**Keywords:** Statins, Gut microbiota, Dysbiosis, Bile acids, Short-chain fatty acids, Type 2 diabetes mellitus, Pregnane X receptor

## Abstract

**Background:**

Statins are a class of therapeutics used to regulate serum cholesterol and reduce the risk of heart disease. Although statins are highly effective in removing cholesterol from the blood, their consumption has been linked to potential adverse effects in some individuals. The most common events associated with statin intolerance are myopathy and increased risk of developing type 2 diabetes mellitus. However, the pathological mechanism through which statins cause these adverse effects is not well understood.

**Results:**

Using a murine model, we describe for the first time profound changes in the microbial composition of the gut following statin treatment. This remodelling affected the diversity and metabolic profile of the gut microbiota and was associated with reduced production of butyrate. Statins altered both the size and composition of the bile acid pool in the intestine, tentatively explaining the observed gut dysbiosis. As also observed in patients, statin-treated mice trended towards increased fasting blood glucose levels and weight gain compared to controls. Statin treatment affected the hepatic expression of genes involved in lipid and glucose metabolism. Using gene knockout mice, we demonstrated that the observed effects were mediated through pregnane X receptor (PXR).

**Conclusion:**

This study demonstrates that statin therapy drives a profound remodelling of the gut microbiota, hepatic gene deregulation and metabolic alterations in mice through a PXR-dependent mechanism. Since the demonstrated importance of the intestinal microbial community in host health, this work provides new perspectives to help prevent the statin-associated unintended metabolic effects.

**Electronic supplementary material:**

The online version of this article (doi:10.1186/s40168-017-0312-4) contains supplementary material, which is available to authorized users.

## Background

Statins are a group of highly prescribed therapeutics used as first-line agents for primary and secondary prevention of coronary artery disease. Statins reduce the risk of heart disease by lowering blood cholesterol levels through two mechanisms. The first mechanism involves inhibiting the enzyme 3-hydroxy-3-methyl-glutaryl-coenzyme A reductase [1], which catalyses a rate-limiting step in the mevalonate biosynthetic pathway. This pathway generates scaffold intermediates not only for the synthesis of cholesterol but also for the production of other biologically important molecules such as heme, vitamin K, coenzyme Q10 (CoQ10), steroid hormones or bile acids (BAs) [2]. By reducing the concentration of cholesterol in the hepatocytes, the second mechanism involves statins inducing the expression of low-density lipoprotein (LDL) receptors, which, in turn, enhances the clearance of LDL cholesterol (LDL-C) from the blood. In addition to lowering LDL-C levels, statins have been reported to have anti-inflammatory and immunomodulatory activities, and there is mounting evidence that statins reduce growth and virulence of a number of bacterial pathogens [3–5].

Although statin’s effectiveness in treating hyperlipidaemia has been evaluated in numerous randomised trials, controversy remains about their safety, mainly due to the well-documented adverse effects in some individuals [6, 7]. The most common events associated with statin intolerance include myopathy, myalgia and, less frequently, myositis and rhabdomyolysis [8]. It has been suggested that the statin-associated muscle damage is caused by mitochondrial dysfunction. This is based on the lower bioavailability of CoQ10 and heme in statin-treated patients [9]. These are two important end products of the mevalonate pathway that function as electron carrier and radical-scavenging antioxidants in the respiratory electron chain and oxidative phosphorylation pathways [9]. Nevertheless, although complementing statin therapy with CoQ10 supplements seems a logical option to prevent the incidence of myotoxicity, there is contradictory evidence as to whether statin-induced myopathy can be alleviated with CoQ10 supplementation [10, 11]. Statins also increase the risk of type 2 diabetes mellitus (T2DM) [12]. This is likely to be linked to interfering with insulin signalling and glucose homeostasis [13]. T2DM is a metabolic disorder associated with insulin resistance, with an initial increase in insulin secretion, however, over time beta cell death and insulin insufficiency. Although T2DM has multifactorial aetiology, recent association studies have highlighted the importance of perturbations in the gut microbiota as a T2DM-contributing factor [14–16]. Thus, T2DM patients present a characteristic gut microbial profile depleted in butyric acid-producing bacteria that may contribute to developing this condition [14]. Butyric acid is a short-chain fatty acid (SCFA) that is derived from the fermentation of non-digestible carbohydrates by saccharolytic gut microbes. This compound is one of the most important metabolites produced by intestinal bacteria based on its multiple beneficial effects on host health. These include the regulation of several processes affected by statin treatment, such as lipid and glucose metabolism and muscle homeostasis [17].

The aim of this study was to investigate the impact of statin treatment on the composition of the mouse gut microbiota and the development of T2DM. The data demonstrate that long-term exposure to statins perturbs the mouse gut microflora and upregulates transcription in the liver of fasting-related genes through a pregnane X receptor (PXR)-dependent mechanism. Importantly, this is the first study to demonstrate that statin therapy results in profound changes in the composition of the bacterial community in the gut.

## Results

### Altered host physiology in response to statins

For this study, two statins were used for their different physiochemical properties to minimise the drug selection bias: atorvastatin is lipophilic while pravastatin is hydrophilic. Clinical data demonstrates that the pharmacokinetic properties are also quite different: atorvastatin has a longer half-life (15–30 versus 1.3–2.8 h), is absorbed more slowly (time to maximum plasma concentration 2–3 versus 0.9–1.6 h) and undergoes first-pass metabolism resulting in reduction of its bioavailability (12 versus 18%) [18].

C57BL/6J wild-type female mice were treated for 12 weeks with either pravastatin, atorvastatin or without treatment (vehicle), in combination with a normal diet (ND) or a high fat diet (HFD). A faster body weight gain trend was observed in the atorvastatin cohort, resulting in significant differences at week 7. Interestingly, this pattern was even more remarkable when both atorvastatin therapy and HFD were combined since significant differences were observed at week 1. To a lesser extent, a similar trend was observed in the ND-pravastatin group. Of note, statins did not affect the overall caloric intake consumption levels, suggesting that the induced differences in body weight were likely through alterations to metabolic factors in the host. Unexpectedly, levels of total cholesterol in the plasma of statin-treated mice were equivalent to those of the control cohort after 12 weeks of treatment (Additional file 1: Figure S1A-D).

Insulin sensitivity was assayed by glucose tolerance test and by measuring fasting blood glucose level at week 11. Unlike the mice fed with HFD, the glucose tolerance test did not show statistical evidence of impaired glucose tolerance in the ND-statin-treated mice (Additional file 1: Figure S1E). On the other hand, fasting blood glucose levels trended to increase in the ND-statin groups compared to the control (ND-vehicle). Although we did not get statistical evidence for this effect, it has been reported that T2DM and non-diabetic patients receiving statin therapy showed elevated fasting plasma glucose levels [19]. As expected, mice fed with a HFD showed significantly higher levels of fasting glucose than the control group (ND-vehicle). Combination of atorvastatin and HFD exacerbated the effect of HFD alone, since the atorvastatin-HFD group exhibited a median glucose concentration over 240 mg dL^−1^ (244 mg dL^−1^), which has been previously suggested as indicative of diabetes in mice [20]. Conversely, pravastatin therapy reversed the diet-induced impaired glucose assimilation during fasting (Additional file 1: Figure S1F).

### Statin treatment alters gut microbiota

We next investigated whether statin therapy affected the gut microbiota. For this purpose, a *16S rRNA* gene sequencing approach was carried out. After performing the quality controls, 2,323,449 raw sequences were obtained in 29 samples plus one technical control with an average length of 414 bp. Using the next-generation sequencing analysis pipeline of the SILVA 16S rRNA gene database (SILVAngs 1.3) [21] with standard settings, 99.82% of the reads could be mapped to the SILVA taxonomy (release version 123) which is resolving down to the genus level (95% identity threshold) for initial taxonomic profiling. Remaining reads could not be classified, usually caused by PCR artefacts such as chimeras.

**Fig. 1:**
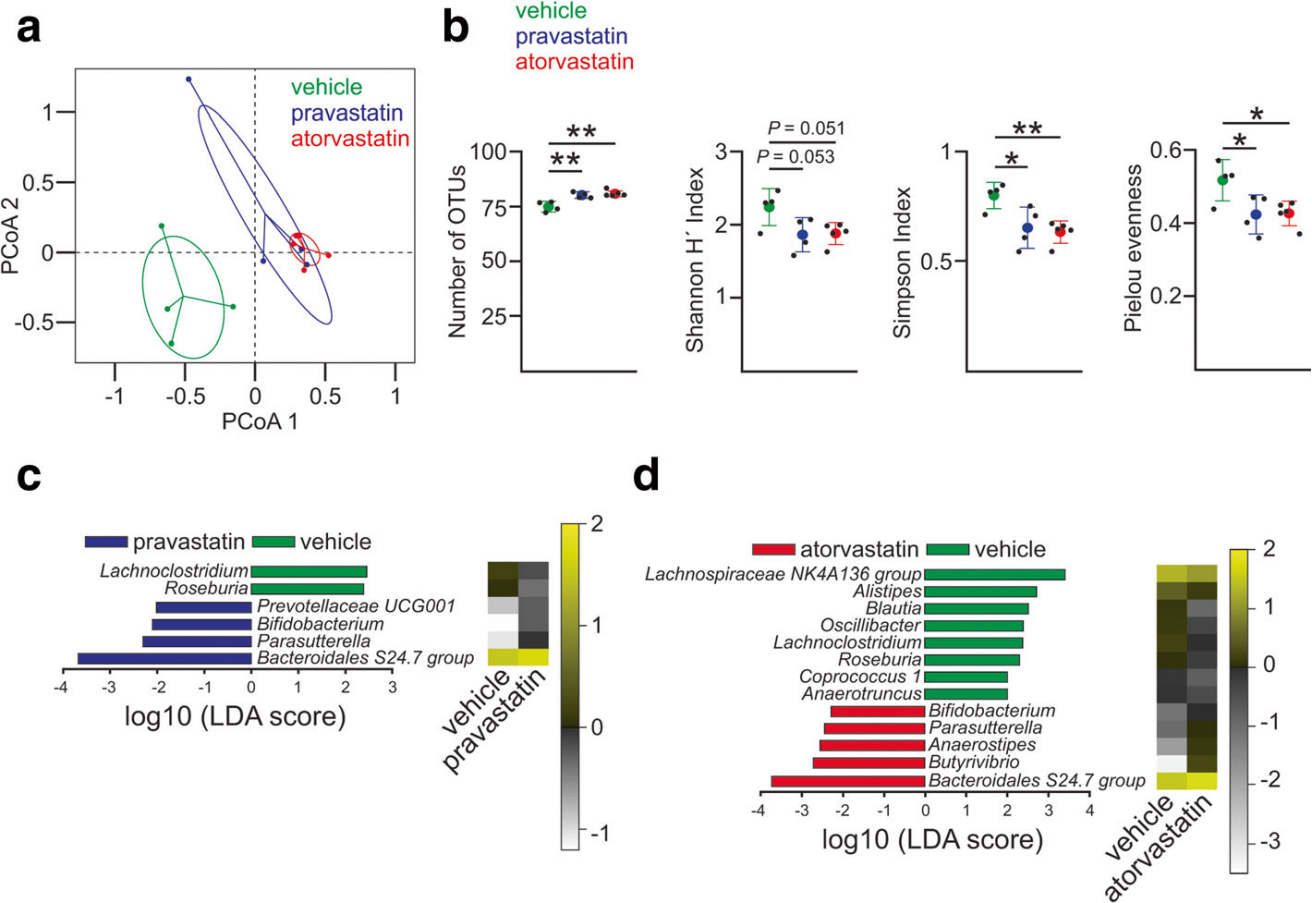
Changes in the gut microbial composition in response to ND-statins. **a** Principal coordinates analysis projection plot showing ordination of the samples using the Bray-Curtis dissimilarity matrices. *Dots* correspond to one individual within each control (vehicle, *green*) and statin (pravastatin, *blue*; atorvastatin, *red*) cohorts combined with normal diet. *Lines* connect each sample to the centroid of the corresponding treatment. *Ellipses* limits represent 95% confidence for the group centroid. **b** Biological diversity was quantified by the Shannon and Simpson indices of diversity as implemented in the R package vegan [67]. The higher the Shannon and Simpson indices, the greater the diversity. Pielou evenness (*J*) was calculated as *J* = *H*′ / log(*S*), where *H*′ is the Shannon index and log(*S*) is the natural logarithm of the number of OTUs. The lower the Pielou index, the less even the community. Each *black point* represents one individual, and the *coloured dots and brackets* show the mean and standard deviation (SD), respectively. The effect of the treatment was evaluated by one-way ANOVA followed by Dunnett’s post hoc test. *P ≤ 0.05; **P ≤ 0.01. **c**, **d** Distinctive gut microbiota composition associated with statin consumption revealed by linear discriminant analysis (*LDA*). Graphs represent the LDA scores of the differentially abundant OTUs associated with the pravastatin (**c**) or atorvastatin (**d**) treatment. Taxa enriched in the gut of mice treated with statins are represented with negative LDA scores. Positive LDA scores represent OTUs enriched in the control cohort (vehicle). Heatmaps on the *right* show the averaged relative abundance (log_10_ transformed) of the discriminative OTUs for the indicated treatments

Principal coordinates analysis (PCoA) shows statistical significant differences in beta diversity between the three groups (permutational multivariate analysis of variance (PERMANOVA): pseudo *F* = 6.71, *P* < 0.01, *R* ^2^ = 0.5732; analysis of similarity (ANOSIM): global *R* = 0.5465, *P* < 0.01). Based on the sum of squares (57.32% explained by statin treatment), this analysis suggests that statin therapy had a strong effect in structuring the composition of the gut microbial community (Fig. 1a).

### Characterisation of the statin-associated gut microbiota

In agreement with a previous report [22], our data indicated that the predominant phyla in the control cohort (ND-vehicle) were *Firmicutes* and *Bacteroidetes* with an average relative abundance in the amplicon pool of 58.5 and 39.6%, respectively (Additional file 1: Figure S2A-B). Statin treatment resulted in a decrease in community diversity as shown by both Shannon and Simpson indices. Richness was slightly increased while the evenness of the species distribution diminished, suggesting that the gut microbiota was dominated by a limited number of species. Accordingly, the overall high-level taxonomic composition of the gut was affected with this imbalance being more pronounced after atorvastatin treatment (Fig. 1b, Additional file 1: Figure S2A-B).

To determine those relevant operational taxonomic units (OTUs) that consistently represent the observed changes in the gut microbial composition after statin therapy, we carried out pairwise comparisons between the statin-treated and the control groups using the linear discriminant analysis effect size (LEfSe) algorithm [23]. Six significantly different OTUs were obtained that differentiated between the vehicle and the pravastatin groups and 13 OTUs between the vehicle and the atorvastatin cohorts (Fig. 1c, d). When both statin-treated groups were compared, only two OTUs were significantly different, suggesting that both statins impacted the composition and structure of the gut microbiota with a similar degree (Additional file 1: Figure S2C). Statin therapy triggered a large enrichment within the phylum *Bacteroidetes*. This proliferation was not division-wide but was due to the expansion of the family *Bacteroidales S24.7*. Consequently, this taxonomic group largely dominated the gut of the statin-treated cohorts. Conversely, statin therapy was characterised by a marked reduction in the abundance of many gram-positive OTUs within the phylum *Firmicutes* belonging to the families *Lachnospiraceae* and *Ruminococcaceae*, constituents of the *Clostridium clusters XIVa* and *IV*, respectively. Members of both clusters of non-pathogenic commensal *Clostridia* are spore formers and synthesise butyric acid as the end product of carbohydrate fermentation, a metabolite that has been demonstrated to be essential for the maintenance of host health and homeostasis [17]. Since spore-forming bacteria could potentially be more resilient to environmental stresses, the reduction of this group of microbes may suggest that statins are inducing changes in the physicochemical environment of the gut. Importantly, enrichment of *Bacteroidetes* over *Firmicutes* in the intestinal microbiota after statin treatment may suggest a shift from butyrate towards acetate, lactate and succinate production. Interestingly, similar changes in the overall gut microbial profiles have been previously reported for diet-induced diabetes-sensitive mice [24].

Compared to the ND cohort, high fat feeding resulted in an enrichment of the phylum *Bacteroidetes* over *Firmicutes*, triggered by a dramatic depletion of several OTUs within the families *Lachnospiraceae* and *Ruminococcaceae* and a pronounced proliferation of bile-tolerant microbes of the genus *Bacteroides* (Additional file 1: Figure S3). When statin therapy was combined with HFD, the gut microbial composition did not substantially differ from the microbiota resulting from fat intake alone and the microbiological diversity of the intestine was not further affected (PERMANOVA: pseudo *F* = 1.73, *P* = 0.053, *R* ^2^ = 0.216) (Additional file 1: Figure S4A).

### Altered SCFA metabolism in statin-treated cohorts

**Fig. 2:**
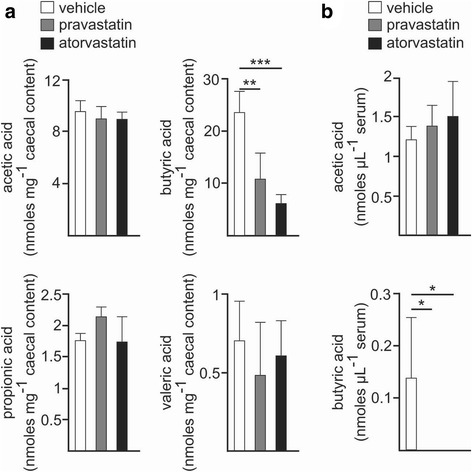
Statins induce a functional dysbiosis. **a**, **b** Levels of the indicated SCFA in the caecum (**a**) or serum (**b**) of wild-type mice fed with ND and treated with statins (pravastatin, *grey*; atorvastatin, *black*) or without treatment (vehicle, *white*). *Barplots* represent the mean ± standard deviation (SD) calculated from at least three biological replicates. *P ≤ 0.05; **P ≤ 0.01; ***P ≤ 0.001; one-way ANOVA and pairwise comparisons by Dunnett’s post hoc test

To evaluate if the observed changes in the gut microbiota in response to statins resulted in altered SCFA metabolism, the SCFA composition was analysed in the faecal content of the caecum and the serum of the control and statin-treated ND cohorts. In agreement with the observed taxonomic profiles, statin treatment resulted in a dramatic reduction in the production of butyric acid whereas levels of acetic, propionic and valeric acids remained unaltered in both statin-treated and control groups. Accordingly, butyric acid was only detected in the serum of control mice, whereas acetic acid concentration did not significantly differ in all tested groups (Fig. 2a, b). These altered fermentation profiles suggest that statin therapy results in a functionally defective gut microbiota.

Because of the high proportions of gram-negative OTUs in the statin-treated mice, we assessed if the statin-associated gut microbiota produces more lipopolysaccharide (LPS) endotoxin. For this purpose, we quantified the antigen load derived from intestinal bacteria by measuring the concentration in the serum of LPS-binding protein (LBP) by enzyme-linked immunosorbent assay (ELISA). LBP is an acute-phase protein that is overexpressed in the liver in response to the presence of LPS in the blood [25]. This experiment revealed no overt differences between the different statin-treated groups with respect to the control cohort (ND-vehicle) (Additional file 1: Figure S5).

### The statin-associated gut metagenome is enriched with genes involved in energy metabolism

To better understand the biological significance of the alterations in the intestinal microbiota composition induced by statins, we investigated the metabolic features of the resulting bacterial communities using Tax4Fun [26]. Tax4Fun is a prediction tool for inferring the potential metabolism of a given bacterial community from a SILVA-annotated OTU table, using precomputed Kyoto Encyclopedia of Genes and Genomes (KEGG) pathway reference profiles [26, 27]. Statin-induced gut remodelling resulted in a predicted metagenomic pattern similar to that previously described for diet-induced obesity in mice (Additional file 1: Figure S6) [28]. Compared to the control cohort (ND, vehicle), enrichment in genes for energy metabolism (glycolysis/gluconeogenesis, pentose phosphate pathway and phosphotransferase system) involved in the production of reducing equivalents and ATP and the uptake of carbohydrates was observed. In contrast, a significant depletion of genes encoding KEGG metabolic pathways related to bacterial motility (bacterial chemotaxis and flagellar assembly) was associated with statin therapy. These cellular structures for locomotion and sensing are widespread in bacteria and respond to environmental changes in highly dynamic habitats. These results highlight the prevalence of non-motile microorganisms in the statin-associated intestinal community and may suggest a loss of the physicochemical heterogeneity in the gut microenvironment following this treatment.

In agreement with the observed taxonomic composition, the predicted metabolic potential of the gut microbiota resulting from HFD intake was not significantly affected by statin therapy (Additional file 1: Figures S7-S8).

### Statins modulate the size and composition of the BA pool in the gut

**Fig. 3:**
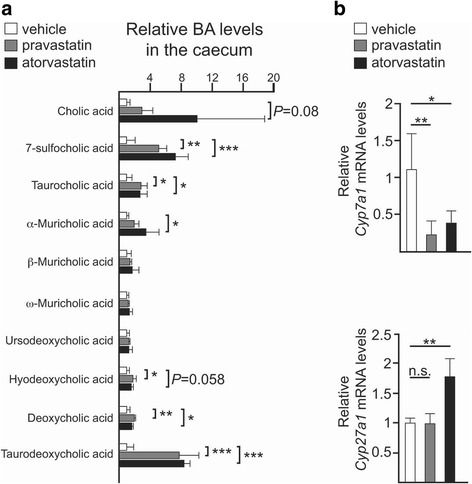
Statins alter the overall composition of the bile acid pool in the gut. **a** Relative levels of the indicated primary and secondary bile acids in the faecal content of the caecum of mice control (*white*) or mice treated with pravastatin (*grey*) or atorvastatin (*black*) and fed with ND. *Bars* represent the mean ± SD calculated from at least four biological replicates. **b** Relative expression in the liver of *Cyp7a1* and *Cyp27a1* by qPCR. Data are represented as the mean ± SD determined from at least three biological replicates. *n.s.* non-significant; *P ≤ 0.05; **P ≤ 0.01; ***P ≤ 0.001; one-way ANOVA and pairwise comparisons by Dunnett’s post hoc test

The size and composition of the BA pool strongly influence the structure of the gut community, since conditions that disrupt bile acid excretion or absorption are linked to gut dysbiosis [29, 30]. In order to probe for a molecular explanation for the statin-induced gut dysbiosis, we decided to evaluate if the observed statin-mediated alterations in the gut microbiota were related to changes in the BA pool in the gut. Analysis of the BA signature in the intestine revealed an enrichment of primary BAs in both ND-statin-treated cohorts. The result of this experiment demonstrated larger relative levels of α-muricholic and cholic acids as well as its sulphate conjugate 7-sufocholic acid (7-SCA), accumulated in the gut of atorvastatin- and, to a lesser extent, of the pravastatin-treated mice, suggesting altered regulation of the synthesis of BAs. We also noted increased levels of taurocholic acid in the gut of both statin-treated groups, suggesting that statin-associated gut microbiota is depleted in taurine-degrading bacteria. Interestingly, an increased concentration of taurine-conjugated species has also been observed in the gut of both antibiotic-treated and germ-free rodents [31, 32]. Atorvastatin treatment also triggered a subtle increase in the secondary bile acid deoxycholic acid and, more remarkably, its taurine derivative taurodeoxycholic acid, suggesting a higher 7α-dehydroxylase activity in the statin-associated intestinal microbiota. Hyodeoxycholic acid, another secondary BA, was found to be slightly increased in the gut of both statin-treated mice. By contrast, no differences were observed in the levels of β-muricholic acid, ω-muricholic acid or ursodeoxycholic acid (Fig. 3a).

To determine whether statin treatment affected BA production, we examined the expression in the liver of two major regulatory enzymes of BA synthesis—*Cyp7a1* (cytochrome P450, family 7, subfamily a, polypeptide 1) and *Cyp27a1* (cytochrome P450, family 27, subfamily a, polypeptide 1)—by quantitative polymerase chain reaction (qPCR) [33]. Expression of *Cyp7a1* was strongly reduced in both statin-treated cohorts while *Cyp27a1* was found to be upregulated in the atorvastatin group only (Fig. 3b).

### Statins alter gene expression in the liver

The synthesis and transport of BAs is modulated by the activity of several nuclear receptors including farnesoid X receptor (FXR), peroxisome proliferator activated receptor alpha (PPARα) and PXR as well as by the gut microbiota [31, 33]. To determine the molecular mechanism responsible of the deregulation of both *Cyp7a1* and *Cyp27a1* genes, we analysed the activity of FXR, PPARα and PXR by measuring through qPCR the transcription levels in the liver of some of their target genes.

**Fig. 4:**
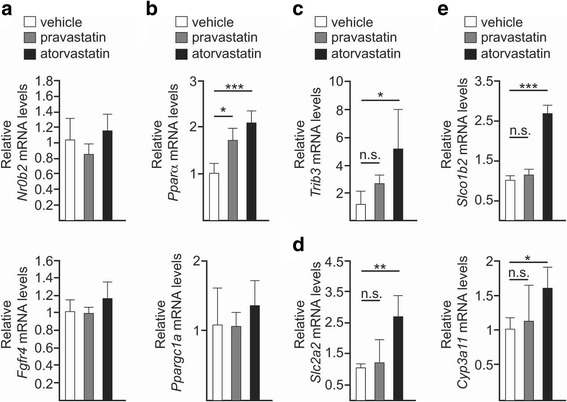
Statins affect the expression in the liver of genes related with lipid and glucose metabolism. **a**–**e** qPCR analysis of the indicated genes in the liver of wild-type control (vehicle, *white*) and statin-treated (pravastatin, *grey*; atorvastatin, *black*) mice fed with ND. *Barplots* represent the mean ± SD determined from at least three biological replicates. *n.s.* non-significant; *P ≤ 0.05; **P ≤ 0.01; ***P ≤ 0.001; one-way ANOVA followed by Dunnett’s post hoc test

Statin therapy did not affect the expression of *Nr0b2* (nuclear receptor subfamily 0, group B, member 2), which encodes the small heterodimer partner (SHP) protein, suggesting that the hepatic FXR pathway was not activated [34]. Expression of FGFR4, another gene product involved in FXR-mediated regulation of *Cyp7a1*, was also not affected by statins (Fig. 4a).

By contrast, atorvastatin and, to a lesser extent, pravastatin therapies resulted in the transcriptional activation of *Pparα*, whereas the transcription rate of its coactivator peroxisome proliferative activated receptor gamma, coactivator 1 alpha (*Ppargc1a*) remained constant (Fig. 4b). Apart from modulating BA metabolism, PPARα is a major regulator of genes involved in lipid homeostasis in the liver [35]. Interestingly, PPARα also governs expression in hepatocytes of *tribbles pseudokinase 3* (*Trib3*), a putative protein kinase that promotes insulin resistance in mice and humans [36, 37]. Notably, *Trib3* transcript increased in both statin-treated mice, the largest effect being observed in the atorvastatin group (Fig. 4c). Finally, we investigated the expression of *Slc2a2* (solute carrier family 2, member 2), which encodes the major glucose transporter of the hepatocytes. We found *Slc2a2* expression to be over 2.5-fold higher in the atorvastatin group compared to the control cohort (Fig. 4d).

On the other hand, we observed the expression of two PXR-targeted genes—*Slco1b2* (solute carrier organic anion transporter family, member 1b2) and *Cyp3a11* (cytochrome P450, family 3, subfamily a, polypeptide 11), to be increased in atorvastatin-treated mice (Fig. 4e). This result is in agreement with a previous study proving that statins activate PXR [38].

### Statins drive gut dysbiosis and metabolic alterations through a PXR-dependent mechanism

We further addressed if activation of PXR by statins could drive the observed gut dysbiosis and metabolic abnormalities. We postulate that if this is the case, the genetic elimination of PXR would result in no overt differences in the physiological response to statins between non-treated and statin-treated mice. Therefore, we subjected a previously described *Pxr* ^*−/−*^ knockout mouse line [39] to statin therapy, using the same experimental conditions and reagents as in our wild-type mouse study.

Compared to the vehicle cohort, statin therapy neither affected the body weight gain trend nor increased fasting blood glucose concentration. Interestingly, atorvastatin-treated mice exhibited a significantly lower caloric intake profile. Of note, as occurred in wild-type mice fed with HFD, pravastatin trended towards lower fasting glucose levels in *Pxr* ^*−/−*^ mice, suggesting that this effect is PXR-independent. Plasma total cholesterol remained unaltered in all tested cohorts (Additional file 1: Figure S9).

Statin-induced changes in the composition of the gut microbiota were attenuated by genetic deletion of *Pxr* (Additional file 1: Figure S10A-B). We noticed a subtle reduction of *Bacteroidetes* and a reciprocal increase of *Firmicutes* (Additional file 1: Figure S10B). PERMANOVA analysis showed significant differences between group centroids (pseudo *F* = 3.4285, *P* < 0.01, *R* ^2^ = 0.3636), indicating that statin therapy induced significant differences in the overall composition of the gut microbial communities (Additional file 1: Figure S10A). In deep contrast to wild-type mice, only 36.36% of the sum of squares was explained by the effect of the statin therapy, indicating that most of the variance remained unexplained and may be due to other factors or random events. To determine if PXR influenced the response to statin therapy of the gut microbiota of wild-type and *Pxr* ^*−/−*^ mice, we carried out a multivariate analysis on the Bray-Curtis dissimilarity matrix of the combined dataset (PERMANOVA, model response as a function of treatment: pseudo *F* = 4.027, *P* < 0.01, *R* ^2^ = 0.1598; genotype: pseudo *F* = 7.76, *P* < 0.001, *R* ^2^ = 0.1540; interaction of treatment and genotype: pseudo *F* = 6.279, *P* < 0.001, *R* ^2^ = 0.2493) (Additional file 1: Figure S11A). Pairwise comparisons between the distances of the different group treatments indicated that the impact of statins on the composition and structure of the gut microbiota was more significant in wild-type mice (Additional file 1: Figure S11B-C).

Evaluation of the microbial diversity in the gut indicated a moderate diminution in the number of OTUs and the evenness of the community after statin therapy (Additional file 1: Figure S10C). LEfSe analysis demonstrated fluctuations in the abundance of the *Lachnospiraceae NK4A136 group*, as a discriminative feature of both statin-treated groups (Additional file 1: Figure S10D). As expected, these oscillations in the abundance of gram-positive bacteria did not significantly alter the serum concentration of the endotoxin-related marker LBP (Additional file 1: Figure S12). Likewise, the predicted metagenomic profiles of the gut microbial communities did not show differences between statin-treated and non-treated groups as large as those observed in wild-type mice (Additional file 1: Figure S13).

Similarly, statin therapy did not result in substantial differences in the production of SCFA by the gut microbiota between treatments, except for a reduction in the levels of acetic acid in the atorvastatin-treated group only (Additional file 1: Figure S14A). Since acetic acid stimulates the release of the hormone that regulates appetite [40], diminished production of acetic acid by the gut microbiota of atorvastatin-treated mice may explain the low food intake profile of this group. Nevertheless, since baseline levels of acetic acid in serum were not consequently reduced (Additional file 1: Figure S14B), this may also reflect a switch from production to utilisation of acetic acid by the gut microbiota.

**Fig. 5:**
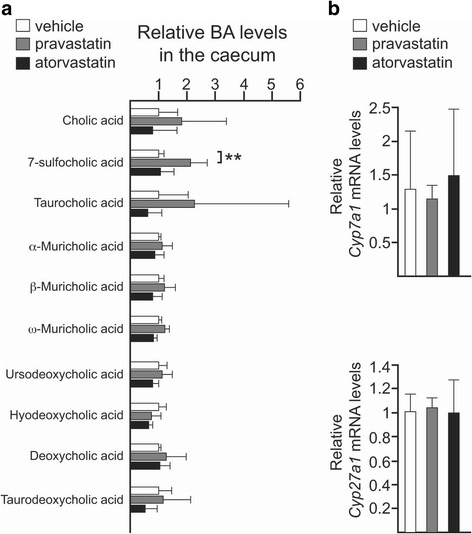
PXR activity regulates the changes in the BA pool of the gut induced by statins. **a** Relative levels of the indicated primary and secondary bile acids in the gut of *Pxr* ^*−/−*^ mice control (*white*) or mice treated with pravastatin (*grey*) or atorvastatin (*black*) and fed with ND. *Bars* represent the mean ± SD calculated from at least four biological replicates. **b** Relative expression in the liver of *Cyp7a1* and *Cyp27a1* by qPCR. *Barplots* represent the mean ± SD determined from three biological replicates. **P ≤ 0.01; one-way ANOVA followed by Dunnett’s post hoc test

Compared to wild-type mice, statins did not result in large changes to the primary BA pool in the gut of *Pxr* ^*−/−*^ mice and, accordingly, no differences in the relative levels of *Cyp7a1* or *Cyp27a1* transcripts were observed (Fig. 5). BA profiling revealed the sulphate-conjugated BA 7-SCA to be specifically increased in the pravastatin group (Fig. 5a), suggesting that pravastatin can modulate the activity of other pathways that control bile acid sulfation such as the constitutive androstane receptor (CAR) [41].

Host gene expression in *Pxr* null mice was not substantially altered by statin therapy (Additional file 1: Figure S15). Like wild-type mice, relative abundance of *Fgfr4* (fibroblast growth factor receptor 4) and *Ppargc1a* messenger RNA (mRNA) was not affected by statins. On the other hand, statin-mediated transactivation of *Slco1b2* and *Pparα* was abolished in the absence of PXR. Conversely, *Slc2a2* mRNA transcript was increased in the atorvastatin-treated group, although compared to wild-type mice, the extent of the increase was more marginal. Intriguingly, we observed that statins downregulated basal gene expression levels of *Nr0b2* and *Cyp3a11* in *Pxr* null hepatocytes. This suggests that statins can bind to different receptors that, in the absence of PXR, suppress both *Nr0b2* and *Cyp3a11* expression. Thus, downregulation of *Nr0b2* is consistent with statins acting as ligands of vitamin D receptor (VDR) [42]. Interestingly, it has been hypothesised that statins, or some of their derived metabolites, could be VDR agonists [43].

## Discussion

### Effect of statins on the host physiology

We have demonstrated that statin therapy drives a profound remodelling in the gut microbiota, hepatic gene deregulation and metabolic alterations in mice through a PXR-dependent pathway. This provides the first empirical evidence of statin-associated secondary effects via an off-target mechanism. Although the major observed effects of statins on host physiology were driven by PXR activation, other nuclear receptors may be selectively activated by different statins [38]. Thus, the amelioration of the glycemic state during fasting of HFD-pravastatin wild-type cohort and pravastatin-treated *Pxr* ^*−/−*^ mice and the higher levels of the sulphate-conjugated BA 7-SCA in the *Pxr* ^*−/−*^ mice receiving pravastatin may suggest that pravastatin also activates CAR in vivo [41, 44]. Interestingly, differential affinity of pravastatin and atorvastatin to CAR and PXR may explain the contrasting diabetogenic character of both drugs [45].

We did not observe a statin-induced reduction of plasma cholesterol levels. This unexpected result may be explained by the fact that among all plasma lipoproteins, statins specifically target the levels of LDL-C, which, compared to humans, is underrepresented in mice [46]. Given that a previous study has shown that statins and other PXR ligands can also induce hepatic cholesterol synthesis in mice [47, 48], it would be reasonable to conclude that statin-mediated reduction of a low-abundant lipoprotein could be confounded by our long-term study.

### Effect of statins on gut microbiota

In contrast to our findings, a previous short-term study did not report appreciable changes in the abundance of specific members of the intestinal microbiota after statin therapy except for a subtle increase of *Lactobacillus* spp. [49]. These differences may be explained by the nature of the experimental design employed between the two studies. Catry and colleagues monitored a subset of bacteria within the gut microbiota by qPCR using species-specific primer pairs after 1 week of statin therapy [49]. In contrast, we carried out a complete *16S rRNA* gene sequencing approach using an optimised primer set following 12 weeks of statin treatment.

We observed that statin treatment had an impact on the diversity of the bacterial community, as shown by a drop in the Shannon and Simpson diversity indices. Interestingly, the Pielou evenness index provided evidence that statin therapy made the intestinal community less even and, in agreement, the *Bacteroidales S24.7 group* dominated the gut of statin-treated mice. Statin-mediated gut remodelling negatively impacted gram-positive taxa mostly within the phylum *Firmicutes*. This resulted in a microbiome composition with a higher capacity for energy production and with metabolic features similar to those previously described for diet-induced obesity-linked gut microbiota [28]. The convergence in the potential functional profiles between statin treatment and HFD may contribute to the higher weight gain observed in the statin-treated cohort.

The likely function of the statin-associated modulation in microbiota genes was predicted by using Tax4Fun. A limitation of this approach is the number of non-model microorganisms that have their genome sequenced and included in the KEGG database. Additionally, since the compositional analysis is carried out analysing the *16S rRNA* profiles, we are excluding any contribution coming from viral or eukaryotic DNA. Finally, this analysis only provides the composition for genes, so we are only predicting the potential functionality of these genomes. Thus, although potential biases exist, we consider our predictions to be reliable since we obtained a functional profile for the HFD-associated community similar to that previously reported using a conventional shotgun metagenomic sequencing approach (Additional file 1: Figure S7) [28]. Based on these functional predictions, the statin-associated microbiota likely exhibits an increased capacity for energy harvest. In addition, as already reported in T2DM patients [14], the statin-associated gut microbiota exhibited an impaired production of butyric acid, indicating functional dysbiosis. Since the known role of SCFAs in regulating gut barrier function and lipid, glucose and cholesterol metabolism, changes in the SCFA profile can impact the physiology of the host and contribute to the development of the T2DM phenotype [17].

### Effect of statins on BA metabolism

We found the BA pool enlarged in the gut of wild-type mice treated with statins, suggesting a deregulation in the synthesis and/or transport of these metabolites. Accordingly, *Cyp27a1* mRNA was upregulated in the liver of the atorvastatin-treated cohort, partly explaining the altered BA profile observed in the gut. In a previous short-term study consisting of 7 days of atorvastatin therapy, Fu and colleagues described high expression of genes involved in the synthesis and transport of BAs, including both *Cyp7a1* and *Cyp27a1* [50]. We observed the expression of *Cyp7a1* reduced in our long-term study, which may be a result of the persistent activation of PXR over time [51]. But importantly, the deregulation of BA metabolism at early stages after the beginning of treatment would contribute to the progressive selection of BA-tolerant microorganisms in the gut. In addition, BAs can activate mechanisms of virulence to favour the establishment of chronic infecting pathogens [52]. Interestingly, commensal components of the human gut belonging to the order *Bacteroidales* secrete antimicrobial proteins and type VI secretion systems that antagonise the growth of both prokaryotic and eukaryotic cells [53, 54]. Thus, it is tempting to speculate that the increase in the *Bacteroidales S24.7* group could be triggered by the BA activation of these competition mechanisms to increase the fitness of these microorganisms in the highly populated and competitive gut environment. A similar molecular mechanism involving changes in the BA profile may contribute to the gut dysbiosis recently reported among proton pump inhibitor users [55], since those therapeutics also induce PXR-mediated transcriptional activity [56].

### Effect of statins on hepatic gene regulation

Interestingly, statin therapy altered expression in hepatocytes of three different genes related to lipid and glucose homeostasis: *Pparα*, *Trib3* and *Slc2a2*. PPARα is the master regulator of lipid metabolism in the liver by targeting the expression of genes involved in fatty acid uptake and intracellular trafficking, lipid deposition and, mostly, β-oxidation [35]. *Pparα* is activated during fasting to enhance the formation of ketone bodies through hepatic oxidation of fatty acids. Fasting also upregulates *Trib3* expression in the liver through a PPARα-dependent mechanism, resulting in glucose output through inhibition of the hepatic insulin signalling [57, 58]. Since the expression of both *Pparα* and *Trib3* in hepatocytes is induced during fasting conditions [57, 59], it is tempting to speculate that the liver responds to statin therapy by stimulating glucose output and increasing fatty acid β-oxidation. Thus, a prolonged fasting-like response could contribute to insulin resistance, mitochondrial oxidative stress and liver damage [58, 60]. Furthermore, statin-mediated expression of *Pparα* may underlie the molecular mechanism by which fibrates, a group of strong PPARα agonists used to treat dyslipidaemia [61], amplify the risk of severe muscle damage on statins [62, 63].

Earlier work has demonstrated that overexpression of *Trib3* in the liver increases hepatic glucose output and blood glucose concentration by inhibiting the activity of the serine/threonine kinase AKT [57]. In addition, *Slc2a2* transcription is stimulated by glucose [64]. Taken together, these data may explain the trend towards increased levels of fasting blood glucose observed in the ND-statin-treated mice and may suggest that statin therapy induces a hyperglycaemic state that could contribute to the development of T2DM.

In this scenario, the influence of the statin-induced dysbiotic gut microbiota in contributing to an aberrant gene expression profile is intriguing. It has recently been reported that perturbing the homeostatic intestinal microbiota with antibiotics induces an epigenetic and transcriptional reprogramming of host cells [65]. Similarly, a role for the statin-associated dysbiotic gut community in the altered hepatic gene expression profiles observed could also be possible. In addition, our work provides interesting questions such as how the host can shape the gut microbiota by activating specific nuclear receptors. Further experiments involving faecal transplants of statin-treated donors into normal mice will help to elucidate the influence of the statin-associated gut microbiota on host homeostasis.

## Conclusions

**Fig. 6:**
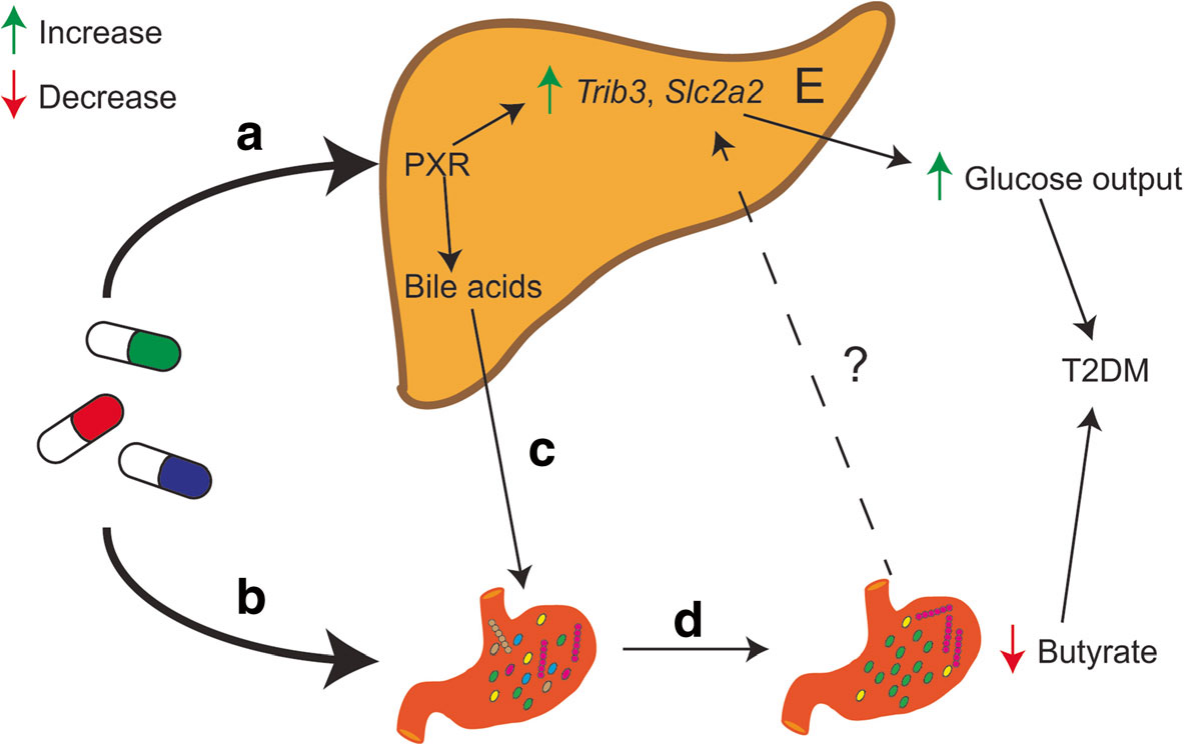
A PXR-dependent mechanism underlies the observed statin-associated secondary effects. Proposed mechanism by which statins may increase the risk of developing T2DM. Activation of PXR in the liver by statins and/or their derived metabolites deregulates BA metabolism (**a**). Based on the antimicrobial properties of statins (**b**) and BAs (**c**), the structure and diversity of the gut microbiota is affected. Progressive selection of BA- and statin-tolerant microbes alters the potential metabolism of the gut microbiota and results in a dysbiotic community defective in the production of butyric acid (**d**). Lower production of butyrate by the gut microbiota together with the aberrant expression in the liver of genes related to glucose metabolism (**e**) may predispose the host to develop new onset of T2DM

We have provided genetic evidence for the hepatic activation of a PXR-dependent mechanism underlying the observed secondary effects of statins (Fig. 6). It will be of interest to examine whether statin therapy also triggers PXR activation and gut dysbiosis in humans. Taken together, our work expands on the existing knowledge of the physiological effects of statins and advances the case for exploring new and attractive strategies such as the use of BA sequestrants and/or PXR, TRIB-3 or PPARα antagonists to help treat and prevent the statin-associated unintended effects.

## Methods

### Mice treatment

Female C57BL/6J mice were obtained at 8 weeks of age from the Animal Resource Centre (Canning Vale, WA, Australia) and were allowed to acclimatise for 1 week. *Pxr* ^*−/−*^ knockout mice were previously described [39] and purchased from Taconic Biosciences. Age-matched mice (five to six per cage) were randomly distributed into the different treatment groups and allowed to eat and drink ad libitum. The normal rodent chow consisted of 14.5% protein, 4% fat and 7% crude fibre, providing 13 MJ kg^−1^ of digestible energy (Specialty Feeds). HFD was a modification of the AIN93G composed of 36% total fats and 20% protein, and the dietary carbohydrate is sucrose. Total fats provide for 59% of calories, and the digestible energy is 22.8 MJ kg^−1^ (SF03-002; Specialty Feeds, Glen Forest, WA, Australia). All food was weighed before and after supply to each cage, and HFD was changed every other day to maintain freshness. A 10 mg kg^−1^ solution/suspension of pravastatin or atorvastatin was prepared fresh and administered daily via gastric gavage at approximately the same time each day, at noon. The vehicle consisted of sterile water. The duration of the treatment lasted 12 weeks. During treatment, mice were weighed weekly and blood was collected weekly through tail bleeds for arterial blood after a 5–6-h fasting period: started in the morning shortly after the start of the light phase. Blood was collected with capillaries coated with heparin, and the plasma and red blood cell fractions were stored separately at −80 °C. Following completion of the treatments and prior to culling, blood was collected through cardiac puncture and stored as above. Faecal material was collected from the caecum, snap-frozen and stored at −80 °C.

### Measurement of blood glucose concentration

Plasma samples from fasted mice stored at −80 °C were assayed for glucose concentration with the Glucose Colorimetric Assay Kit (Cayman Chemical Company, Ann Arbor, MI, USA) according to the manufacturer’s instructions. Plasma samples were diluted 1:10 with an assay diluent prior to using the standard protocol, utilising a standard curve run in parallel. The absorbance of 514 nm was read on an EnSpire Multimode Plate Reader (PerkinElmer Australia, Melbourne, VIC, Australia).

### Oral glucose challenge test

The oral glucose challenge test was performed at week 11 of the treatment according to the optimised parameters described by Andrikopoulos and colleagues [66]. The mice were fasted for 5–6 h followed by administration of a fixed amount of glucose in solution (50 mg) into the gut, based on 2 g kg^−1^. A microvolume of blood was taken from the tail prior to the bolus administration of glucose and then following at 10, 20, 30, 40 and 60 min. Glucose levels were determined immediately with a glucometre (OneTouch VerioIQ; LifeScan, Inc., Chesterbrook, PA, USA).

### Cholesterol and LBP analysis

Levels of circulating cholesterol were determined using the Cholesterol Quantitation Kit (SIGMA, MAK043) as per the manufacturer’s instructions. Serum LBP levels were determined using a commercial ELISA kit (Hycult Biotech, HK205-02).

### DNA isolation and *16S rRNA* taxonomic profiling

Samples (100 mg of caecal content) were homogenised with zirconia/silica beads (0.1 mm diameter) in a bead beater before proceeding with the isolation of total DNA using the QIAamp Fast DNA Stool Mini Kit (Qiagen, 51604) as the manufacturer’s instructions with minor modifications.

To identify and subtract the sequences of contaminating DNA generated during the extraction procedure, we included negative controls during this technical step. *16S rRNA* gene library preparation and sequencing were carried out at LGC Genomics GmbH. Briefly, PCR amplification of the *16S rRNA* gene (forward primer: 341F *5′-NNNNNNNNNNTCCTACGGGNGGCWGCAG* and reverse primer: 785R *5′-NNNNNNNNNNTGACTACHVGGGTATCTAAKCC*) was carried out for 30 cycles using the following parameters: predenaturation at 96 °C for 2 min, 96 °C for 15 s, 50 °C for 30 s and 70 °C for 90 s. About 20 ng amplicon DNA of each sample carrying different barcodes was pooled and purified with one volume of AMPure XP beads (Agencourt) to remove primer dimer and other small mispriming products, followed by an additional purification on MinElute columns (Qiagen). Illumina libraries were constructed using the Ovation Rapid DR Multiplex System 1-96 (NuGen) and sequenced on an Illumina MiSeq desktop sequencer using V3 chemistry. Data were processed using the SILVAngs pipeline (https://www.arb-silva.de/ngs) [21]. Briefly, reads shorter than 350 aligned nucleotides or sequences with low quality (reads with more than 2% of ambiguities or 2% of homopolymers) were excluded for further processing. After alignment, identical reads were removed and unique reads clustered (OTUs) and the reference read of each cluster identified using a local nucleotide BLAST search against the non-redundant version of the SILVA SSU Ref dataset (release 123; http://www.arb-silva.de) with standard settings.

### Data analysis

Analysis of the gut microbial community was carried out using R (version 3.2.4, http://www.r-project.org). OTU tables were prefiltered to remove OTUs with low counts (only OTUs representing at least 1% of the total community were selected for downstream analysis) and normalised by rarefaction using the R package vegan [67]. OTU tables were rarefied to 51,671 and 4713 sequences to analyse the effect of statin therapy in the wild-type and *Pxr* ^*−/−*^ datasets, respectively. The combined dataset including wild-type and *Pxr* ^*−/−*^ samples was rarefied to 4711 sequences. Data structure was analysed by PCoA using the Bray-Curtis dissimilarity matrices. To test the differences between the centroids of the predefined groups, we performed PERMANOVA as implemented in the function *adonis* of the R package vegan using 10,000 permutations. Multivariate spread homogeneity assumption was confirmed by a permutation-based statistical test. Significant differences between treatment groups were evaluated using ANOSIM. Treatment groups were considered significantly different if *P* is <0.05.

For linear discriminant analysis, files were prepared in R and LEfSe analysis was computed at the OTU level using Galaxy (http://huttenhower.sph.harvard.edu/galaxy/). For retaining discriminative features, a logarithmic LDA score higher than 2 (absolute value) was applied. Alpha values for Kruskal-Wallis and Wilcoxon tests were less than 0.05.

Prediction of the OTU-associated microbiome was carried out using Tax4Fun [26] and the rarefied OTU tables as input. Taxonomic profiles were normalised by the *16S rRNA* gene copy number and the functional metagenome predicted using KEGG pathway reference profiles precomputed using UProC [68]. To study the effect of the statins on KEGG pathway composition, the relative abundance of each KEGG family gene was normalised to a total number of 25,000 genes.

Statistical comparisons of the mean between groups were performed by one-way ANOVA followed by Tukey’s honestly significant difference (TukeyHSD) or Dunnett’s post hoc test, considering a *P* value ≤0.05 as statistically significant.

### Bile acid and short-chain fatty acid profiling

For BA analysis, samples (≈40 mg of caecal content) were homogenised with acetonitrile using zirconia/silica beads (0.1 mm diameter). After discarding stool particles, the supernatant was evaporated in a vacuum centrifuge and solubilised in a volume of methanol to a final concentration of 1 μL mg^−1^ of gut content. Chromatographic separation was performed on Agilent 1290 Infinity UHPLC using a 150 mm × 2.1 mm internal diameter (i.d.) Phenomenex Kinetex® C18 core-shell column, packed with 2.6-μm particles. HPLC was carried out with mobile phase A (0.1% formic acid in aqueous solution) and mobile phase B (0.1% formic acid in acetonitrile) at a total flow rate of 0.5 mL min^−1^. Gradient program was increased linearly from 5% mobile phase B and 95% mobile phase A to 100% mobile phase B for 9.5 min. Bile acid identities were established in negative ion mode using a mass MSMS instrument (Agilent QTOF 6540) and the following pure standards: cholic acid (C1129, SIGMA), deoxycholic acid (D4297, SIGMA), lithocholic acid (L6250, SIGMA), chenodeoxycholic acid (C1050000, European Pharmacopoeia Reference Standard), cholic acid 7-sulphate (9002532, Cayman Chemical), α-muricholic acid (C1890-000, Steraloids), β-muricholic acid (sc-477731, Santa Cruz), ω-muricholic acid (C1888-000, Steraloids), ursodeoxycholic acid (C1020-000, Steraloids), hyodeoxycholic acid (H0535, TCI), taurocholic acid (sc-220189, Santa Cruz) and taurodeoxycholic acid (15935, Cayman Chemical). Peak integration and analysis was performed using ProFinder (software version B.06.00, Agilent Technologies) and a customised spectral library.

For SCFA profiling, samples were spiked with 5 nmol of ^13^C-sodium acetate (279293, SIGMA) and 5 nmol of 2-ethyl butyric acid (109959, SIGMA) as internal standards and were homogenised in isopropanol. After centrifugation, 1 μL of the supernatant was injected into a HP 6890 Series GC System, equipped with an Agilent 5973 Network Mass Selective Detector in splitless mode. Samples were separated on a Stabilwax®-DA (Shimadzu) column (30 m × 0.25 mm i.d.) coated with a 0.25-μm-thick film. The carrier gas was helium at a flow rate of 1 mL min^−1^. The initial oven temperature of 90 °C was held for 2 min, then increased to 240 °C at 5 °C min^−1^ and maintained for additional 2 min. The temperature of the quadrupole, MS source and inlet were 150, 230 and 250 °C, respectively. Identities and retention times of the SCFA were established using the volatile-free acid mix (46975-U, Supelco). Peaks were automatically integrated using MSD ChemStation (version D.03.00.611). SCFA concentration was estimated using the internal references ^13^C-sodium acetate (for acetic acid) or 2-ethyl butyric acid (for all the others SCFA tested). Data were calculated as nanomoles per microlitre serum or per milligram caecal content from at least three biological replicates within each different group.

### RNA extraction and quantitative RT-PCR

RNA extraction from ≈40 mg of the frozen liver was carried out using the Isolate II RNA/DNA/Protein Kit (Bioline) following the manufacturer’s protocol. To ensure tissue disruption, a homogenisation step using zirconia/silica beads (1 mm diameter) was included. For the synthesis of complementary DNA (cDNA) from a 1.5-μg RNA template, we used the High Capacity cDNA RT Kit (Applied Biosystems) according to the manufacturer’s instructions. Predesigned KiCqStart^®^ Primers: *Cyp7a1* (M_Cyp7a1_1), *Cyp27a1* (M_Cyp27a1_1), *Pparα* (M_Ppara_1), *Trib3* (M_Trib3_1), *Slc2a2* (M_Slc2a2_2), *Ppargc1a* (M_Ppargc1a_2), *Cyp3a11* (M_Cyp3a11_3), *Slco1b2* (M_Slco1b2_1), *Nr0b2* (M_Nr0b2_1), *Fgfr4* (M_Fgfr4_2), *Tbp* (TATA box-binding protein; M_Tbp_1) and *Gusb* (M_Gusb_2) were purchased from SIGMA. For expression analysis, reactions were prepared using the SensiFAST™ SYBR^®^ Lo-ROX Kit (Bioline) and performed on a CFX Connect™ Real-Time PCR Detection System (Bio-Rad). qPCR cycling conditions were 95 °C for 2 min and then 40 cycles of 95 °C for 5 s, 60 °C (61.5 °C for *Nr0b2*) for 10 s and 72 °C for 15 s. Amplicon melting curves were recorded after cycle 40 by a temperature gradient from 65 to 95 °C at 0.5 °C increase every 5 s. The amplification of a single PCR product was also confirmed by agarose gel electrophoresis. Each biological replicate was run in duplicates, and the mean quantification cycles (Cq) of these technical replicates was used in downstream calculations. Expression was determined from at least three biological replicates by the 2^−(ΔCq test sample − ΔCq calibrator sample)^ method [69]. Raw Cq data were normalised using the geometric mean of *Tbp* and *GusB* reference gene expression for each sample. Normalised Cq values (ΔCq) were then subtracted by the calibrator sample. As the calibrator, we used the averaged ΔCq of the biological replicates within the vehicle (control) group.

## Additional file

Additional file 1: Figure S1. Effect of statin therapy and diet on body weight and glucose metabolism. **Figure S2.** Changes in the gut microbiome composition in response to statins of mice fed with ND. **Figure S3.** Changes in the gut microbiome composition in response to high fat diet. **Figure S4.** Statin therapy does not potentiate the diet-induced intestinal dysbiosis. **Figure S5.** Variation of LBP levels in serum in response to statin therapy and diet. **Figure S6.** Metagenome prediction based on the community composition of the gut microbiota of *wild type* mice treated with statins and normal diet. **Figure S7.** Metagenome prediction based on the community composition of the gut microbiota of *wild type* mice treated with statins and high fat diet. **Figure S8.** Metagenome prediction based on the community composition of the gut microbiota of *wild type* mice treated with statins and high fat diet. **Figure S9.** Effect of statin therapy and diet on body weight and glucose metabolism in *Pxr-/-* mice. **Figure S10.** Effect of statin therapy on the gut microbiota of *Pxr-/-* mice. **Figure S11**. Changes in the gut microbial community in response to statins differ based on the activity of PXR. **Figure S12.** Variation of LBP levels in serum of *Pxr-/-* mice in response to statin therapy. **Figure S13.** Metagenome prediction based on the community composition of the gut microbiota of *Pxr-/-* mice treated with statins. **Figure S14.** Production of short chain fatty acid by the gut microbiota of *Pxr-/-* mice treated with statins. **Figure S15.** PXR modulates the changes in gene expression induced by statins. (ZIP 5 mb)

## Abbreviations

7-SCA: 7-sufocholic acid
ANOSIM: analysis of similarity
BA: bile acid
CAR: constitutive androstane receptor
cDNA: complementary DNA
CoQ10: coenzyme Q10
Cq: quantification cycles
Cyp27a1: cytochrome P450, family 27, subfamily a, polypeptide 1
Cyp3a11: cytochrome P450, family 3, subfamily a, polypeptide 11
Cyp7a1: cytochrome P450, family 7, subfamily a, polypeptide 1
ELISA: enzyme-linked immunosorbent assay
Fgfr4: fibroblast growth factor receptor 4
FXR: farnesoid X receptor
GusB: glucuronidase beta
HFD: high fat diet
KEGG: Kyoto Encyclopedia of Genes and Genomes
LBP: lipopolysaccharide-binding protein
LDA: linear discriminant analysis
LDL: low-density lipoprotein
LDL-C: low-density lipoprotein cholesterol
LEfSe: linear discriminant analysis effect size
LPS: lipopolysaccharide
mRNA: messenger RNA
ND: normal diet
Nr0b2: nuclear receptor subfamily 0, group B, member 2
OTU: operational taxonomic unit
PCoA: principal coordinates analysis
PERMANOVA: permutational multivariate analysis of variance
Ppargc1a: peroxisome proliferative activated receptor gamma, coactivator 1 alpha
PXR: pregnane X receptor
qPCR: quantitative polymerase chain reaction
SCFA: short-chain fatty acid
SD: standard deviation
SHP: small heterodimer partner
Slc2a2: solute carrier family 2, member 2
Slco1b2: solute carrier organic anion transporter family, member 1b2
SRA: Sequence Read Archive
T2DM: type 2 diabetes mellitus
Tbp: TATA box binding protein
Trib3: tribbles pseudokinase 3
TukeyHSD: Tukey’s honestly significant difference
UProC: ultrafast protein classification toolbox
VDR: vitamin D receptor
