# Supplementary material for: Statin therapy causes gut dysbiosis in mice through a PXR-dependent mechanism
Source: Microbiome. 2017 Aug 9;5:95. doi: 10.1186/s40168-017-0312-4 (PMC5550934; doi:10.1186/s40168-017-0312-4)
Supplement: Additional file 1: Figure S1. — Effect of statin therapy and diet on body weight and glucose metabolism. Figure S2. Changes in the gut microbiome composition in response to statins of mice fed with ND. Figure S3. Changes in the gut microbiome composition in response to high fat diet. Figure S4. Statin therapy does not potentiate the diet-induced intestinal dysbiosis. Figure S5. Variation of LBP levels in serum in response to statin therapy and diet. Figure S6. Metagenome prediction based on the community composition of the gut microbiota of wild type mice treated with statins and normal diet. Figure S7. Metagenome prediction based on the community composition of the gut microbiota of wild type mice treated with statins and high fat diet. Figure S8. Metagenome prediction based on the community composition of the gut microbiota of wild type mice treated with statins and high fat diet. Figure S9. Effect of statin therapy and diet on body weight and glucose metabolism in Pxr-/- mice. Figure S10. Effect of statin therapy on the gut microbiota of Pxr-/- mice. Figure S11. Changes in the gut microbial community in response to statins differ based on the activity of PXR. Figure S12. Variation of LBP levels in serum of Pxr-/- mice in response to statin therapy. Figure S13. Metagenome prediction based on the community composition of the gut microbiota of Pxr-/- mice treated with statins. Figure S14. Production of short chain fatty acid by the gut microbiota of Pxr-/- mice treated with statins. Figure S15. PXR modulates the changes in gene expression induced by statins. (ZIP 5 mb) [file 40168_2017_312_MOESM1_ESM.zip › Caparros-Martin_Supp_Fig10.pdf]

Supplemental Figure 10.

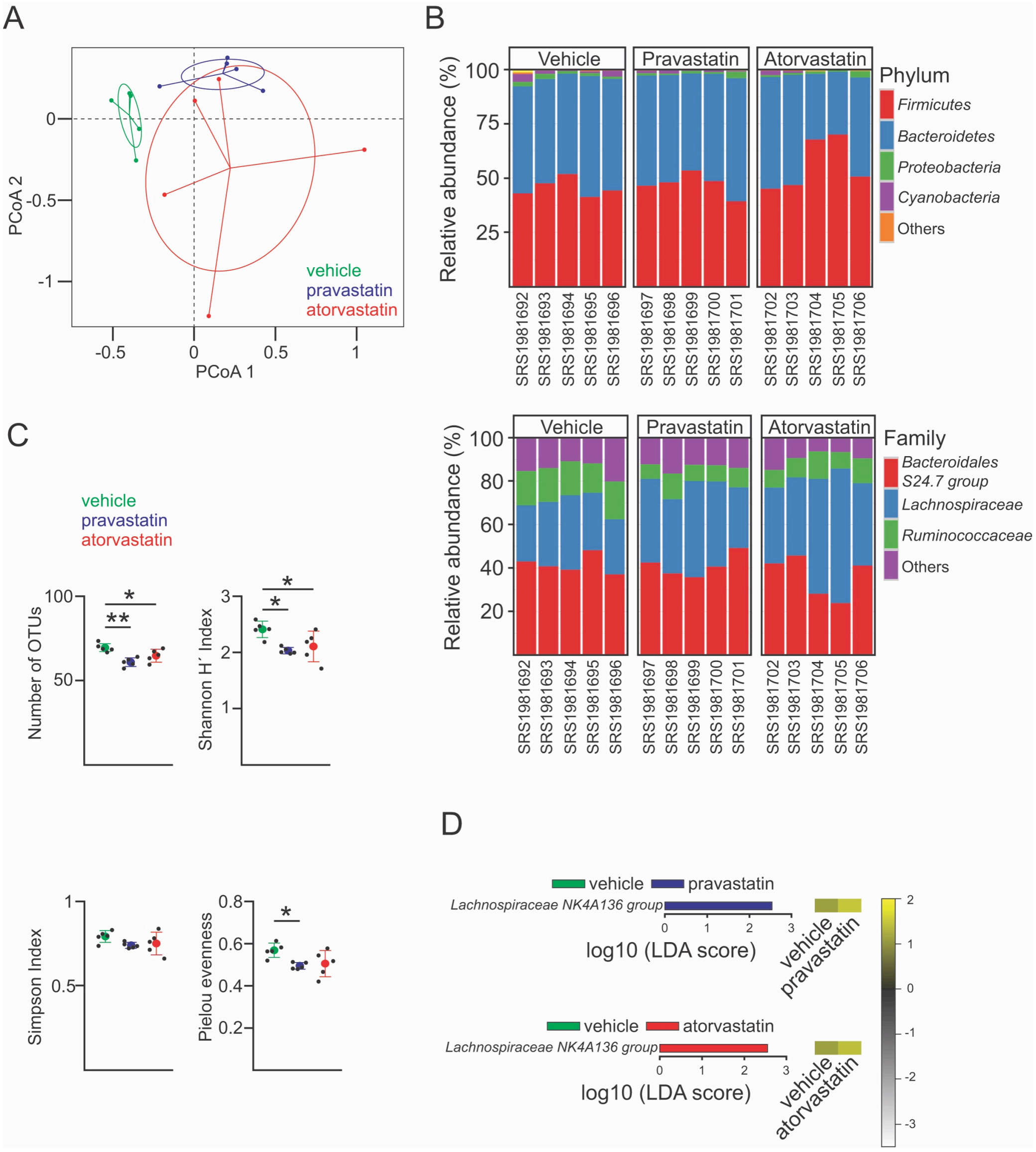

<u>Supplemental Figure 10. Effect of statin therapy on the gut microbiota of *Pxr*$^{-/-}$ mice.</u> **A.** Principal coordinates analysis projection plot showing ordination of the samples using Bray-Curtis dissimilarity matrices. Dots correspond to one individual within each control (vehicle, green) and statin (pravastatin, blue; atorvastatin, red) cohorts combined with normal diet. Lines connect each sample to the centroid of the corresponding treatment. Ellipses limits represent 95% confidence for the group centroid. **B.** Relative microbial taxonomic composition of the gut at phylum (above) and family level (below). Each bar represents one individual. Samples are named with their corresponding SRA accession number. **C.** Biological diversity was quantified by the Shannon and Simpson indices of diversity as implemented in the R package vegan. The

higher the Shannon and Simpson's indices, the greater the diversity. Pielou evenness (*J*) was calculated as $J = H'/log(S)$, where H' is the Shannon index and $log(S)$ is the natural logarithm of the number of OTUs. The lower the Pielou index is, the less even the community. Each black point represent one individual and the coloured dots and brackets show the mean and standard deviation (SD) respectively. *, $P \leq 0.05$; **, $P \leq 0.01$; one-way ANOVA and pairwise comparisons by Dunnett's *post hoc* test. **D.** Distinctive gut microbiota composition associated with statin consumption $Pxr^{-/-}$ mice revealed by Linear Discriminant Analysis (LDA). Graphs represent the LDA scores of the differentially abundant OTUs associated with the pravastatin (above) or atorvastatin (below) treatment. Taxa enriched in the gut of mice treated with statins are represented with negative LDA scores. Positive LDA scores represent OTUs enriched in the control cohort (vehicle). Heatmaps on the right show the averaged relative abundance (log_{10} transformed) of the discriminative OTUs for the indicated treatments.
