# Supplementary material for: Statin therapy causes gut dysbiosis in mice through a PXR-dependent mechanism
Source: Microbiome. 2017 Aug 9;5:95. doi: 10.1186/s40168-017-0312-4 (PMC5550934; doi:10.1186/s40168-017-0312-4)
Supplement: Additional file 1: Figure S1. — Effect of statin therapy and diet on body weight and glucose metabolism. Figure S2. Changes in the gut microbiome composition in response to statins of mice fed with ND. Figure S3. Changes in the gut microbiome composition in response to high fat diet. Figure S4. Statin therapy does not potentiate the diet-induced intestinal dysbiosis. Figure S5. Variation of LBP levels in serum in response to statin therapy and diet. Figure S6. Metagenome prediction based on the community composition of the gut microbiota of wild type mice treated with statins and normal diet. Figure S7. Metagenome prediction based on the community composition of the gut microbiota of wild type mice treated with statins and high fat diet. Figure S8. Metagenome prediction based on the community composition of the gut microbiota of wild type mice treated with statins and high fat diet. Figure S9. Effect of statin therapy and diet on body weight and glucose metabolism in Pxr-/- mice. Figure S10. Effect of statin therapy on the gut microbiota of Pxr-/- mice. Figure S11. Changes in the gut microbial community in response to statins differ based on the activity of PXR. Figure S12. Variation of LBP levels in serum of Pxr-/- mice in response to statin therapy. Figure S13. Metagenome prediction based on the community composition of the gut microbiota of Pxr-/- mice treated with statins. Figure S14. Production of short chain fatty acid by the gut microbiota of Pxr-/- mice treated with statins. Figure S15. PXR modulates the changes in gene expression induced by statins. (ZIP 5 mb) [file 40168_2017_312_MOESM1_ESM.zip › Caparros-Martin_Supp_Fig11.pdf]

Supplemental Figure 11.

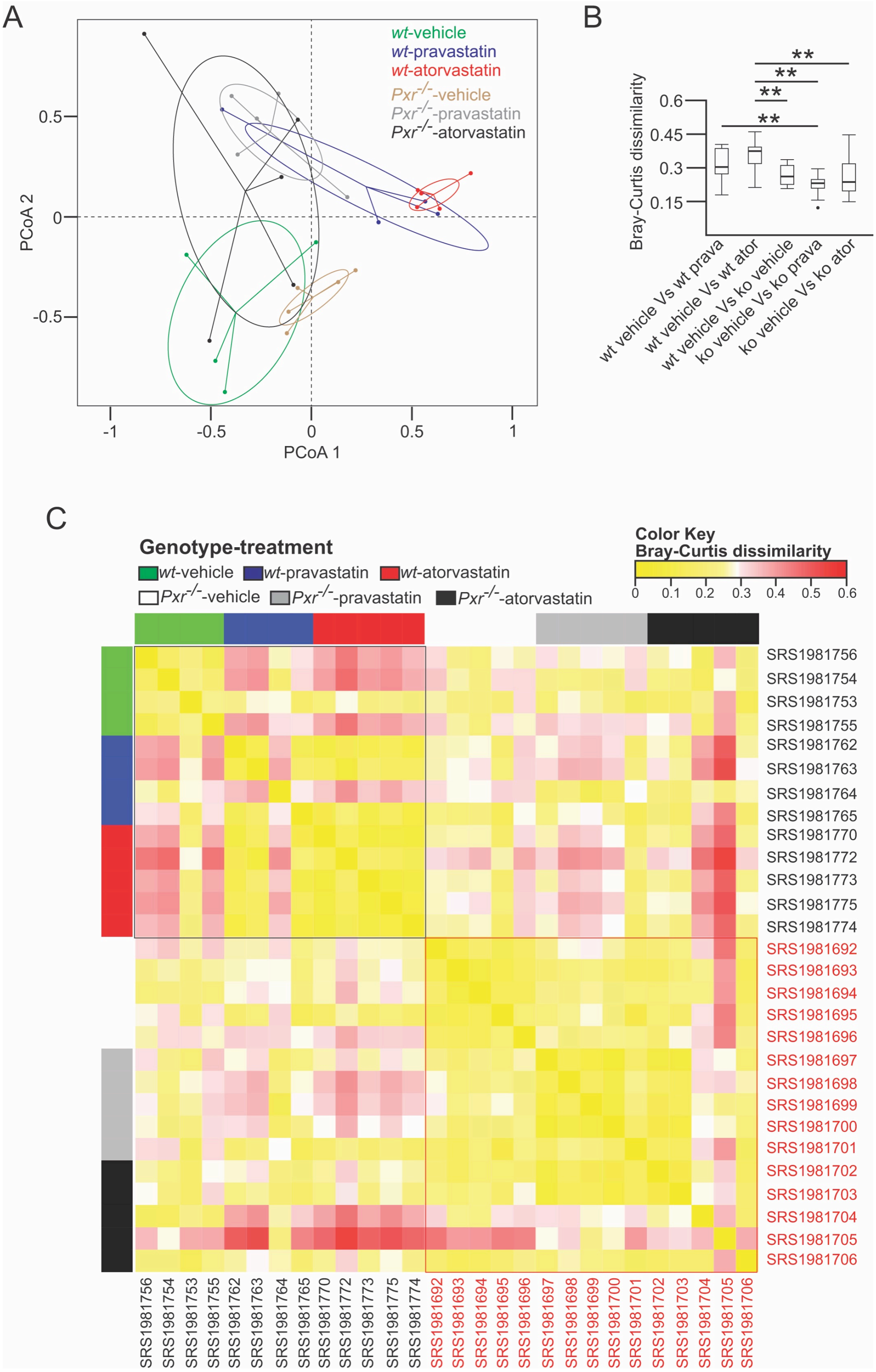

Supplemental Figure 11. Changes in the gut microbial community in response to statins differ based on the activity of PXR. **A.** Principal coordinates analysis projection plot showing ordination of the samples using Bray-Curtis dissimilarity matrices. Dots correspond to one individual within each of the indicated cohorts

fed with ND. Lines connect each sample to the centroid of the corresponding treatment. Ellipses limits represent 95% confidence for the group centroid. **B.** Box plot represents median and quartiles for Bray Curtis dissimilarity distances between the indicated pairwise comparisons of selected groups. **, $P \leq 0.01$; one-way ANOVA and pairwise comparisons by TukeyHSD *post hoc* test. Only statistically significant differences are indicated. **C.** Heatmap showing Bray Curtis dissimilarity distances between samples. Colours in the heatmap correspond to distances as indicated in the colour key. Rectangles delimit the dissimilarity measures among the samples with the same genotype (wild type, black; $Pxr^{-/-}$, red). The different combinations of genotype-treatment are indicated with coloured bars on the left hand side and the top part of the heatmap. Samples are named with their corresponding SRA accession number and coloured accordingly to the mouse genotype (wild type, black; $Pxr^{-/-}$, red).
