# Supplementary material for: Statin therapy causes gut dysbiosis in mice through a PXR-dependent mechanism
Source: Microbiome. 2017 Aug 9;5:95. doi: 10.1186/s40168-017-0312-4 (PMC5550934; doi:10.1186/s40168-017-0312-4)
Supplement: Additional file 1: Figure S1. — Effect of statin therapy and diet on body weight and glucose metabolism. Figure S2. Changes in the gut microbiome composition in response to statins of mice fed with ND. Figure S3. Changes in the gut microbiome composition in response to high fat diet. Figure S4. Statin therapy does not potentiate the diet-induced intestinal dysbiosis. Figure S5. Variation of LBP levels in serum in response to statin therapy and diet. Figure S6. Metagenome prediction based on the community composition of the gut microbiota of wild type mice treated with statins and normal diet. Figure S7. Metagenome prediction based on the community composition of the gut microbiota of wild type mice treated with statins and high fat diet. Figure S8. Metagenome prediction based on the community composition of the gut microbiota of wild type mice treated with statins and high fat diet. Figure S9. Effect of statin therapy and diet on body weight and glucose metabolism in Pxr-/- mice. Figure S10. Effect of statin therapy on the gut microbiota of Pxr-/- mice. Figure S11. Changes in the gut microbial community in response to statins differ based on the activity of PXR. Figure S12. Variation of LBP levels in serum of Pxr-/- mice in response to statin therapy. Figure S13. Metagenome prediction based on the community composition of the gut microbiota of Pxr-/- mice treated with statins. Figure S14. Production of short chain fatty acid by the gut microbiota of Pxr-/- mice treated with statins. Figure S15. PXR modulates the changes in gene expression induced by statins. (ZIP 5 mb) [file 40168_2017_312_MOESM1_ESM.zip › Caparros-Martin_Supp_Fig13.pdf]

Supplemental Figure 13.

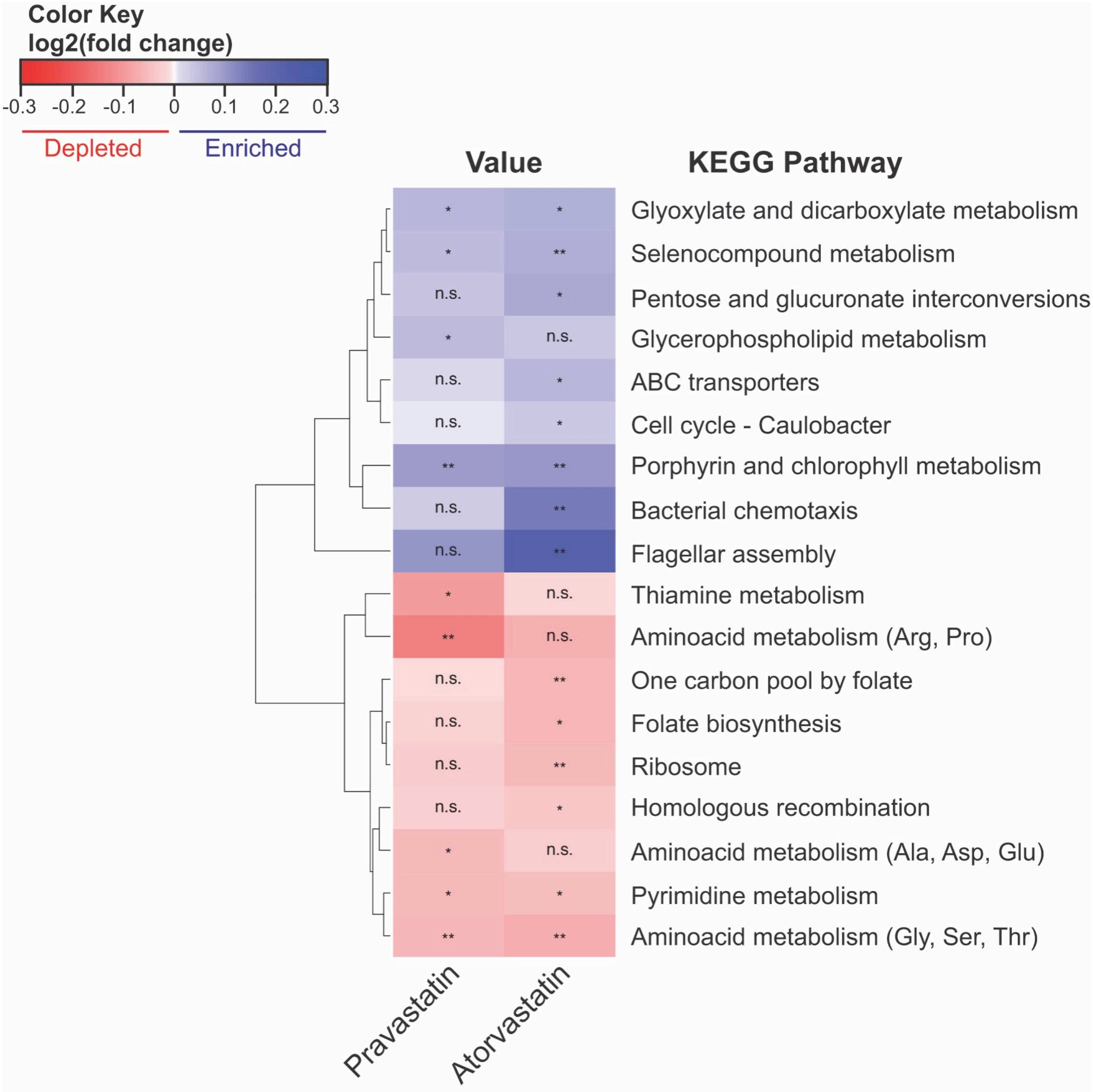


<u>Supplemental Figure 13. Metagenome prediction based on the community composition of the gut microbiota of *Pxr*$^{-/-}$ mice treated with statins.</u> Heatmap showing the KEGG metabolic pathways enriched (blue) or depleted (red) after the indicated statin treatment. Fold changes were calculated for each pathway, by dividing the number of genes of each treatment by the number of genes of the control (vehicle, normal diet) group. Rows represent pathways and columns the different cohorts tested. Pathways were clustered accordingly to the level of similarity after hierarchical clustering calculation. The length of the branch in the dendrograms illustrates the degree of similarity in the response to the treatment of each pathway. The level of significance after performing TukeyHSD *post hoc* test between the represented groups and the control (vehicle, normal diet)

cohorts is indicated within each cell. Only pathways showing significance in at least one of the treatments tested are represented. n.s., non-significant; *, $P \leq 0.05$; **, $P \leq 0.01$.
